# Confidential Audit of Perinatal Mortality in the Republic of Kazakhstan: A Pilot Study

**DOI:** 10.3390/medsci13020077

**Published:** 2025-06-13

**Authors:** Aizada Marat, Zaituna Khamidullina, Svetlana Muratbekova, Kulyash Jaxalykova, Bekturgan Karin, Nazerke Samatova, Umit Usmanova, Madina Sharipova, Aknur Kobetayeva, Milan Terzic, Yesbolat Sakko, Gulzhanat Aimagambetova

**Affiliations:** 1 Department of Obstetrics and Gynecology #1, NJSC “Astana Medical University”, Astana 010000, Kazakhstan; aizada.m.marat@gmail.com (A.M.); zaituna.khamidullina@yandex.ru (Z.K.); madina.sharipova2025@yandex.ru (M.S.); kobetaevaaknur@gmail.com (A.K.); 2 Higher School of Medicine, NJSC Sh. Ualikhanov Kokshetau University, Kokshetau 020000, Kazakhstan; muratbekova.s@mail.ru; 3 Department of Neonatology, NJSC «Astana Medical University», Astana 010000, Kazakhstan; dkk06@mail.ru (K.J.); samatovanazerke@mail.ru (N.S.); yapodarok@inbox.ru (U.U.); 4 Department of Surgery, School of Medicine, Nazarbayev University, Astana 010000, Kazakhstan; milan.terzic@nu.edu.kz; 5 Clinical Academic Department of Women’s Health, CF University Medical Center, Astana 010000, Kazakhstan; 6 Department of Biomedical Sciences, School of Medicine, Nazarbayev University, Astana 010000, Kazakhstan; yesbolat.sakko@nu.edu.kz

**Keywords:** confidential audit, perinatal mortality, neonatal mortality, antenatal mortality, intranatal mortality, stillbirth, Kazakhstan

## Abstract

Introduction: Perinatal mortality is labeled as the loss of fetuses at or beyond 22 weeks of gestation, deaths during labor and delivery, as well as early neonatal deaths. Appropriate medical care provided in the perinatal period is an integral indicator of high-quality medical care. Although developed countries managed to decrease perinatal mortality, it remains high in the developing world. This study aims to perform a confidential audit of perinatal mortality (CAPM) across Kazakhstani maternity hospitals. Methods: A descriptive, observational cross-sectional study was conducted from January 2024 to December 2024. The structure of the underlying causes of mortality in the antenatal, intranatal, and early neonatal periods among different maternity hospitals of the Republic of Kazakhstan was analyzed. Results: A total of 116 cases were assessed: 34 antenatal deaths, 6 intranatal, and 76 early neonatal. Most deaths occurred on the second day post-delivery. The analysis revealed that 93% of cases fell into categories indicating substandard or potentially inadequate care (categories 2 and 3). Intraventricular hemorrhage and sepsis emerged as leading causes of neonatal death. Among antenatal and intranatal deaths, significant proportions were associated with comorbid maternal conditions, insufficient antenatal visits, and inadequate perinatal support. Conclusions: CAPM proves to be a critical tool for identifying systemic gaps and guiding improvements in maternity services without attributing blame to health professionals. Findings underscore that many perinatal deaths could have been avoided with timely, evidence-based interventions across antenatal and neonatal care. Broader implementation and institutionalization of CAPM in Kazakhstan could lead to measurable reductions in perinatal mortality and improvements in maternal/newborn care outcomes. Factors such as preconception planning, improving the health of reproductive-age women, administration of folic acid, and reducing primary cesarean sections could assist in achieving the reduction in the perinatal mortality rate.

## 1. Introduction

The World Health Organization (WHO) defines a perinatal death as “a death occurring at any time from 22 completed weeks (154 days) of gestation and up to seven completed days after birth” [1,2]. This includes stillbirth (intrauterine fetal demise) and early neonatal death (an infant death happening in the first 7 days of life) [2].

Perinatal losses have been inadequately documented, and large deviations exist across the world regions [2,3]. It is estimated that almost 5 million perinatal deaths occur worldwide, with 2 million of these being fetal and 3 million being early neonatal [4,5,6,7,8,9]. Almost all these deaths (99%) occur in low- and middle-income countries (LMICs), and approximately 50% happen outside of healthcare facilities; therefore, they continue to be uncounted [4,5]. Reducing the level of perinatal morbidity and mortality has always been and remains one of the principal concerns of perinatology [1]. Perinatal losses are one of the important indicators reflecting the quality of medical care for pregnant women, infants, and newborns [10]. Moreover, perinatal loss has significant long-term physical, psychological, social, and even financial consequences for parents/families and serious penalties for healthcare professionals and impacts both parents and healthcare workers who care for parents at the time of loss [11,12,13,14,15].

Adequate care and medical assistance provided in the perinatal period are integral indicators of the medical care [16]. Identifying the causes of stillbirth and neonatal death through appropriate diagnostic testing is an important component of quality healthcare for parents and families [17,18]. A better recording and understanding of the causes leading to perinatal deaths is the key factor in addressing the burden of perinatal mortality that occurs each year [19].

The perinatal period is a unique time in terms of its importance for the child’s development and health, and subsequently the health of a nation. This lifetime cannot be compared with any other age period [20]. The exclusivity of the perinatal period is determined by the fact that 99.5% of diseases developed in this period are due to conditions that arise before/during pregnancy and childbirth, and only 0.5% of cases are associated with conditions that are realized during the first week of life.

To improve the quality of care for mothers and newborns and reduce mortality, it is important to study the data associated with pregnancy and labor/delivery, track trends over time, identify risk factors and causes of death, and use this valuable information as learning opportunities and to improve future care [15]. Critically analyzing each death in an impartial, multidisciplinary context has the potential to “tell a story about what could have been done differently to unlock the solutions that should have been available for each woman and baby to prevent perinatal deaths” [6,21]. Audit, combined with feedback from healthcare providers, can change practice and improve health outcomes, especially when followed by an action plan and clear, measurable targets [22].

The confidential audit was implemented into clinical practice in the early 20th century in the United Kingdom (UK) in response to concerns about the high maternal mortality rate at the time, and in 1985, the confidential inquiries were expanded to the national level [23]. Later in 1992, the UK expanded its confidential audit to include perinatal deaths [16]. Confidential audit of perinatal mortality (CAPM) is an anonymous, systematic, and multidisciplinary investigation of perinatal deaths that identifies causes of demise and their preventable factors. CAPM provides a confidential, anonymized, evidence-based context that does not assign blame but takes into account the factors that led to the death of the fetus/newborn [20].

In 2015, the Millennium Development Goals identified maternal health and infant mortality as priorities for United Nations (UN) countries and highlighted the particular need to reduce preventable perinatal mortality, including stillbirths and neonatal deaths worldwide [24]. Although during the past decade, 51 countries worldwide implemented confidential inquiries into maternal mortality, only 17 have performed CAPM [25].

The Republic of Kazakhstan is a middle-income Central Asian country with a population of 20 million [26,27,28]. As of 2022, the birth rate in Kazakhstan increased to 3.05 births per woman, with the annual number of deliveries of approximately 400,000 [26,27]. The Kazakhstani government prioritizes support for maternity and childcare and has allocated a special budget for the “Development of Healthcare 2020–2025” program, with a specific target to decrease maternal and perinatal mortality [26]. Over the past 15 years, there has been a positive trend in reducing perinatal mortality from 22.7 in 2008 to 9.1 in 2023 [29,30]. However, the stagnation of indicators over the past five years requires further analysis of the causes of perinatal mortality to determine the potential ways for improving the situation.

In 2016, at the initiative of the Ministry of Health of the Republic of Kazakhstan and with the technical support of the UN Children’s Fund, an audit of perinatal mortality was introduced in Kazakhstan to analyze the structure and define the causes of perinatal loss [10]. However, since that time, only a few maternity hospitals have followed the CAPM strategy; thus, the opportunities provided by CAPM remain underutilized, and perinatal mortality has not significantly changed and continues to be high. Moreover, the structure and the reasons for perinatal mortality remain underexplored. Thus, this study aims to reinforce CAPM strategies in Kazakhstan and perform a confidential audit of perinatal mortality across Kazakhstani maternity hospitals. The study results will identify a window for improvement of the perinatal care quality and develop proposals for further steps on the way to the reduction in perinatal morbidity and mortality in the Republic of Kazakhstan.

## 2. Materials and Methods

### 2.1. Study Design and Setting

A descriptive, observational cross-sectional study was conducted from January 2024 to December 2024. The structure of the underlying causes of mortality in the antenatal, intranatal, and early neonatal periods in different perinatal centers of the Republic of Kazakhstan was analyzed. The study followed the Strengthening the Reporting of Observational Studies in Epidemiology (STROBE) guideline [31].

### 2.2. Study Instrument and Recruitment

A confidential audit of perinatal mortality is a systematic, multidisciplinary, anonymous investigation of perinatal mortality cases that occurred in a particular medical organization. The investigation determines the number of cases, their causes, and preventable factors that are associated with perinatal mortality.

The study was performed according to the WHO Confidential Inquiry into Perinatal Deaths tool, “Making every baby count: audit and review of stillbirths and neonatal deaths” [6,21]. The audit cycle was carried out by trained experts with the support of the administration and the core members of the steering committee. The audit of perinatal mortality cases was performed by trained obstetrics and gynecology (Z.Kh., A.M., G.A. and A.K.) and neonatology (B.K., K.J., N.S. and U.U.) specialists/experts in CAPM. The collected anonymous records were investigated using the WHO tools to collect data in connection with the regional and/or national healthcare systems context. It included the following 6 steps: (1) identification of cases to be reviewed; (2) collection of information; (3) analysis of information; (4) proposing solutions; (5) implementing solutions; and (6) evaluation of the process and its outcomes [21].

To recruit maternity hospitals into the study, letters were sent to the regional maternity hospitals with an invitation to participate in a confidential investigation of perinatal losses. Medical records of antenatal and neonatal mortality cases and deceased newborns were provided from all regions of Kazakhstan, except for the Mangystau, Ulytau, and Pavlodar regions. A request for medical records was also sent to these areas, but they were not provided. Those regions/medical organizations that confirmed their participation were included in the study. To carry out a confidential audit of perinatal mortality, a Central Commission was created. All medical records on each case of perinatal mortality from maternity hospitals were sent to the Central Commission. After receiving all documents, the particular cases of perinatal mortality were completely anonymized/depersonalized, i.e., the personal data of the patient and medical personnel who participated in the provision of medical care were hidden from the experts; subsequently, all medical documentation underwent the encryption process. Then the perinatal death cases were distributed among the experts for analysis. The CAPM was carried out according to the WHO Confidential Inquiry into Perinatal Deaths tool, “Making every baby count: audit and review of stillbirths and neonatal deaths” [21], by utilizing anonymous questionnaires and analysis of depersonalized medical documentation for each case to establish the causes of death and medical and non-medical factors that led to the death of the fetus or newborn [21].

### 2.3. Cases Selection and Definitions

The study included cases of antenatal, intranatal, and early neonatal death: antenatal fetal death (stillbirth) from 22 weeks of gestation; intranatal fetal death; newborns who died in the neonatal period (the first 28 days of life); and both fetal/newborn genders. All ethnic groups living in the territory of the Republic of Kazakhstan were included. Thus, fetuses demised at a gestational age of ≥22 weeks and newborns in the early neonatal period (0–168 h from birth) with a gestational age of ≥22 weeks and a body weight of 500.0 g or more were included.

The following exclusion criteria were applied: stillborn (in the ante- or intranatal period) at a gestational age of ≥22 weeks with congenital defects incompatible with life; deceased newborns in the early neonatal period (0–168 h from birth) at a gestational age of ≥22 weeks with a body weight of 500.0 g or more with critical defects incompatible with life; deceased newborns in the early neonatal period (0–168 h from birth) at a gestational age of ≥22 weeks with a body weight of 500.0 g or more with confirmed genetic abnormalities [21].

There are four levels of the volume and quality of healthcare services: from level 0, when optimal care was provided but the case could not have been prevented, to level 3, when the care provided was suboptimal but the death was preventable. Mortality category definitions used for CAPM are presented in Table 1.

Risk factors that influence the fatal outcome are classified according to the time period of their influence (during pregnancy, childbirth, or after childbirth) and are divided into (a) factors related to a woman, family and social conditions; (b) factors related to the availability of medical care; (c) factors related to the volume and quality of medical care; and (d) other factors (counseling, mutual understanding with medical personnel, and diagnostics) [21].

### 2.4. Ethical Considerations

The study was conducted in compliance with the Declaration of Helsinki and approved by the Astana Medical University Review Ethics Committee (protocol reference №17 19.02.2024). Waiver of informed consent has been granted due to the anonymized data analysis and retrospective nature of the study.

### 2.5. Statistical Analysis

Descriptive statistics for sociodemographic and clinical characteristics were calculated for all categorical variables using frequencies and percentages. For numeric variables, medians and interquartile ranges were calculated.

Bivariate analysis was performed by stratifying by mortality type—antenatal and intranatal. Pearson’s chi-squared test was used to find associations of the sociodemographic and clinical characteristics with mortality type. A two-sided *p*-value < 0.05 was considered statistically significant. For numeric variables, medians and interquartile ranges were calculated. Statistical analyses and graph plotting were performed using Stata, version 18.5 (StataCorp, LLC, College Station, TX, USA).

## 3. Results

A confidential investigation of 116 cases of perinatal mortality, including 34 cases of antenatal mortality, 6 of intranatal mortality, and 76 of early neonatal mortality, was performed in this study. This number constitutes 3.2% of the overall perinatal mortality in Kazakhstan in 2024.

### 3.1. Analysis of Antenatal and Intranatal Mortality

Overall, 40 cases of antenatal and intranatal mortality were investigated in this CAPM study (34 cases of antenatal mortality and 6 of intranatal mortality). Most cases were represented by the Kazakh ethnic group—80% (Table 2). Eighty-five percent of all women were in committed relationships, had completed secondary education, and were from middle-income families. Out of all antenatal and intranatal mortality cases (40), 60% of mothers had concomitant conditions affecting their pregnancy course, and 45% had not had preconception counseling (Table 2).

Out of antenatal and intranatal mortality cases, 82% of women were registered for pregnancy follow-up before 12 weeks of gestation, and 62% had four or more visits to a gynecologist for pregnancy care. Notably, only 45% of women took folic acid during the first trimester of pregnancy.

All women with hypertensive disorders during pregnancy were taking antihypertensive medications (Table 2). Remarkably, 18% of pregnancies reported were complicated with oligohydramnios and 5% with polyhydramnios. Regarding the timing of delivery, 62% of pregnancies resulted in preterm delivery (vaginal or cesarean).

According to mortality categories, it was revealed that all subjects with perinatal losses were classified into categories 2 and 3. In the group of antenatal losses, 47% of subjects were classified into category 2, and the remaining 53% were classified into group 3. In the group of intranatal losses, 83% were classified into category 2 and 17% into category 3 (Table 3).

**Figure 1:**
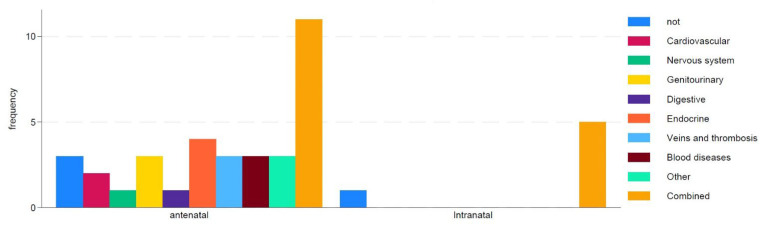
Impact of maternal concomitant diseases on type of death.

Only 10% of women with antenatal and intranatal losses did not have chronic conditions, while 40% had combined diseases (Figure 1). Endocrine system diseases were registered in 10% of cases, a history of venous diseases and thrombotic events in 8%, and cardiovascular diseases in 5% of women.

Twenty-eight percent of women with antenatal and intranatal losses had a history of surgical interventions on the uterus, 5% had undergone surgical interventions on the cervix, and 2% had undergone surgical interventions on the ovaries. The analysis of other confounding factors and their impact on antenatal and perinatal mortality is presented in Supplementary Files (Supplementary File S1).

### 3.2. Analysis of Neonatal Mortality

Overall, 76 cases of neonatal mortality were analyzed in this study. In the majority of cases, the death occurred on day 2 of life (after delivery) (Table 4). Most of the neonatal mortality cases occurred with male newborns—63% vs. 37% of female newborns (Table 4). The median birthweight was 1016.0 g, and the median gestational age at delivery was 27 weeks. Notably, 14% of newborns were small for gestational age, and 87% were born prematurely. Forty-nine percent of cases were without antenatal corticosteroid treatment, and 42% of cases had complete maternal antibiotic prophylaxis.

**Figure 2:**
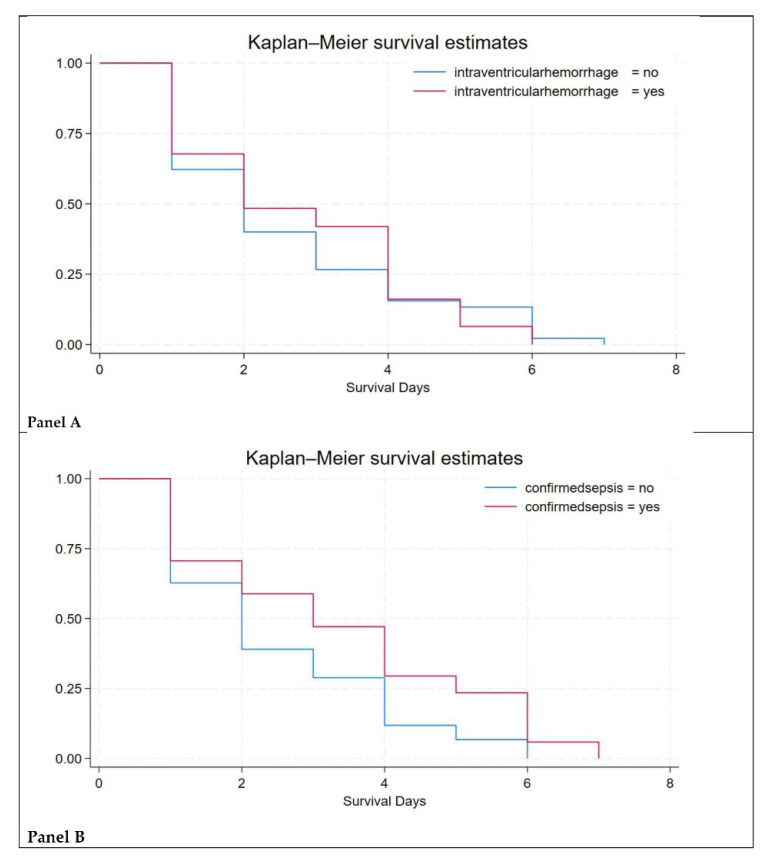
Survival estimates. Figure legend: Panel (**A**)—survival estimates in newborns with intraventricular hemorrhage cases; Panel (**B**)—survival estimates in newborns with sepsis.

In this study, intraventricular hemorrhage (IVH) and sepsis were found to be the major causes of neonatal mortality (Figure 2).

**Figure 3:**
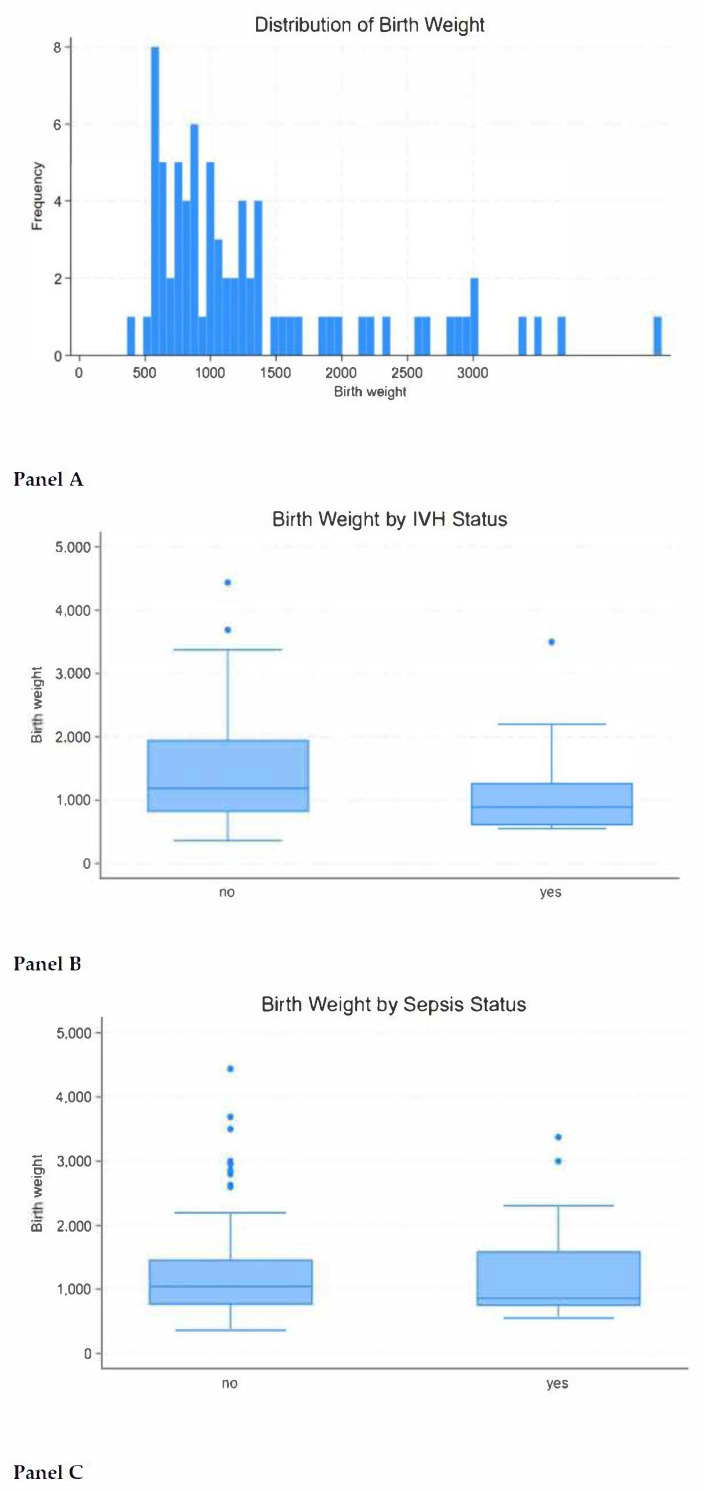
Impact of birthweight on neonatal mortality. Figure legend: Panel (**A**)—birthweight distribution; Panel (**B**)—correlation of birthweight with mortality due to intraventricular hemorrhage; Panel (**C**)—correlation of birthweight with mortality due to sepsis.

Negative correlation was observed between birthweight and mortality: the lower the birthweight, the higher the risk of death from IVH and sepsis (Figure 3 and Supplementary File S2).

The cases of neonatal losses were divided into the same categories as antenatal, intranatal, and neonatal cases, depending on the level of suboptimal care. The majority, 89%, of subjects were classified into categories 2 and 3: In the IVH group, 90% of cases, and in the sepsis group, 88% (Table 3). The remaining 11% were classified into category 1.

## 4. Discussion

A confidential audit of perinatal mortality has a significant impact on pregnancy and neonatal care [21]. It provides a structured, non-punitive approach for understanding the causes and contributing factors of fetal and neonatal deaths. The CAPM approach matters for the identification of preventable causes of perinatal mortality, has the potential to improve clinical practice, promotes accountability and learning, and informs public health strategies [21,32]. Since some drawbacks in perinatal care still exist in the Kazakhstani healthcare system, this study aims to perform a confidential audit of perinatal mortality across Kazakhstani maternity hospitals.

In this study, an expert assessment of 116 perinatal mortality cases was conducted, of which 29% (34) were registered in the antenatal period, 5% (6) in the intranatal period, and 66% (76) in the early neonatal period (the median is day 2 after birth). The proportion of deaths classified as categories 2 and 3 was the overwhelming majority, 93%, which suggests a low level of perinatal care.

Similar results were obtained in another study, which was conducted in Moldova [33]. According to the compared study results, most cases were classified as suboptimal or not meeting standards, which indicates non-compliance with the standards recommended in national guidelines for obstetric and neonatal practice: in 87.7% of cases, it was assessed as suboptimal (categories 2 and 3) [33].

This study was also compared with a previous CAPM investigation that was performed in Kazakhstan in 2017. In that study, six perinatal centers where cases with a high risk of perinatal pathology are concentrated were selected from different cities: Astana, Aktobe, Taraz, Karaganda, Pavlodar, and Shymkent [10]. The previous study reported 113 cases of perinatal losses that occurred over 9 months of 2017: 80% (91) were registered in the antenatal period, 4% (4) in the intranatal period, and 16% (18) in the early neonatal period [10]. The proportion of deaths that were classified as categories 2 and 3 constituted the vast majority, 82%. The results of the compared study are in line with the current study and show that most mortality cases could have been avoided if proper antenatal and intranatal care were provided. The cases were classified as categories 2 and 3 due to the identification of factors of inadequate care, and the most important of these are professional factors related to the volume and quality of care provided by health workers. Consequently, health workers have insufficient professional capacity in the field of antenatal, intranatal, and postnatal care. Unfortunately, comparison of the current study results with the CAPM performed in 2017 reveals that no improvement in perinatal care was observed during that period.

Inadequate care identified during the study could have been avoided if the following factors were ensured: physiological course of pregnancy and safe childbirth, timely diagnosis of pregnancy and childbirth complications, safe care for women and newborns in the postnatal period, as well as timely diagnosis of emergency conditions in newborns and their adequate therapy. All these factors of insufficient care ultimately lead to an increase in perinatal morbidity and mortality in the periods [10].

Based on the results of this study, opportunities for improving perinatal care were identified. Pregnancy planning and preconception counseling appear to be important contributing factors helping to decrease perinatal mortality. Preconceptional counselling allows for assessment of risk factors and provides care to women with modifiable risk factors, such as smoking, as well as adequately controlling chronic diseases [34]. While planning pregnancy, all chronic conditions should be examined, and regression should be achieved to avoid exacerbation during pregnancy, which will negatively affect the pregnancy outcomes. According to the results of this study, only 10% of the women had a healthy background for pregnancy. At the same time, 40% of the women had concomitant diseases that complicated the perinatal period. Thus, it is necessary to strengthen the work on improving the health of women of childbearing age [26].

Appropriate and timely antenatal care is another important tool to improve pregnancy outcomes. In this study, 82% of pregnant women were registered in antenatal care on time, and 62% had more than four visits during pregnancy. Thus, 38% of the women with perinatal losses who visited antenatal care clinics less than four times during pregnancy are one of the main prospects for preventing perinatal losses. More frequent visits will help to monitor and promptly respond to emerging needs of patients and appearing health issues. To reduce perinatal mortality and create a positive experience of receiving care, it is recommended to use antenatal care models that involve at least eight contacts of the pregnant woman with health workers during the pregnancy course. Antenatal care limited to four visits does not provide women with full care during the antenatal period and, thus, may affect the perinatal outcomes [21,32].

Even though in this study the vast majority of women received timely antenatal care, only 45% of subjects took folic acid during the first trimester. The remaining 55% of cases should be considered as room for improvement in perinatal indicators. According to the data obtained, almost every third woman had surgical interventions on the uterus, mainly repetitive cesarean sections. Hence, obstetricians should pay attention to the rising indications and subsequent rates of primary cesarean sections, which are directly linked to obstetric complications. Therefore, one of the main opportunities to improve perinatal outcomes is the reduction in the primary cesarean section. The next key point to discuss is that 62% of all pregnant women with perinatal losses are delivered prematurely, which increases the risk of perinatal morbidity, mortality, and future disability and is also associated with increased healthcare costs for the country, which are not always justified. Improving antenatal healthcare could help prolong pregnancy to a gestational age closer to term, thus decreasing adverse perinatal outcomes.

### Strengths and Limitations

This study offers several important strengths. First, it stands as one of the earliest comprehensive efforts to apply a confidential audit of perinatal mortality at a national level in Kazakhstan, setting a precedent for future quality improvement initiatives. Second, the use of a multidisciplinary panel of trained experts allowed for nuanced, balanced evaluations, free from institutional bias or punitive intent. Third, the inclusion of maternity hospitals from multiple regions introduced valuable geographic and clinical diversity, enhancing the generalizability of the findings. Fourth, the study adhered to WHO-endorsed methodologies for case classification and audit procedures, lending methodological robustness and enabling comparisons with international data. Fifth, the audit process encouraged a reflective, system-focused analysis of care delivery, identifying both clinical and organizational contributors to perinatal loss.

However, the study also has limitations that should be considered. First, despite its wide reach, the sample may not fully represent all healthcare settings in Kazakhstan due to the low sample size and hesitancy to participate in the audit process. Namely, although invited, not all regional maternity hospitals of the country took part in the confidential audit. This hesitancy could be due to a long-term history of blaming culture and fear of being accused if perinatal mortality cases are disclosed. Second, as a retrospective study, it relied heavily on existing medical documentation, which varied in completeness and could have limited the depth of analysis in certain cases. Third, while expert reviewers followed a structured framework, the process inherently involved subjective interpretation, which may have introduced variability in the categorization of care adequacy. Fourth, the absence of qualitative input from patients, families, or frontline staff means that important contextual or psychosocial factors influencing outcomes were not captured. Fifth, given the observational design, the study can suggest associations between suboptimal care and mortality but cannot definitively establish causality.

## 5. Conclusions

This investigation highlights the practical relevance and diagnostic power of confidential audits when applied to perinatal mortality in Kazakhstan. The process not only shed light on shortcomings in clinical practice but also exposed specific moments where intervention might have altered outcomes. Rather than casting blame, the audit’s purpose was to observe patterns, identify lapses, and suggest where improvements could be made. The data make clear that with better antenatal monitoring, more consistent care during labor, and timely neonatal support, many of these deaths might have been prevented.

Moreover, the audit emphasized gaps in both access to and the quality of care, particularly in managing high-risk pregnancies and extremely preterm deliveries. These findings serve as a strong argument for integrating CAPM more systematically across the country’s maternity system. If used regularly and constructively, the audit process could serve as a foundation for ongoing professional development, institutional learning, and policy refinement.

In the broader picture, reducing perinatal deaths in Kazakhstan will require a sustained commitment, not just to protocols and funding, but to thoughtful, reflective care for women and newborns during one of the most vulnerable periods of life. Factors such as preconception planning, improving the health of reproductive-age women, administration of folic acid, and reducing primary cesarean sections could assist in decreasing the perinatal mortality rate. Moreover, healthcare professionals’ training, integration of the confidential audit with health information systems, and strategies for engaging healthcare administrators in the CAPM could improve maternity hospitals’ engagement with the confidential inquiry into perinatal death.

## Figures and Tables

**Table 1:** Mortality category and definitions used for confidential audit of perinatal mortality.

Category	Definition
**0**	Inadequacy of medical care was not identified
**1**	Inadequate medical care; a different management strategy would not have changed the outcome
**2**	Inadequate medical care; a different management strategy could have affected the outcome
**3**	Inadequate medical care; it would be reasonable to expect that a different management strategy might have affected the outcome

**Table 2:** Descriptive statistics of antenatal and intranatal mortality cases.

Factor	Level	Value	Antenatal	Intranatal	*p*-Value	Test
Total		40	34	6		
Ethnic origin	Kazakh	32 (80%)	26 (76%)	6 (100%)	0.41	Pearson’s chi-squared
	Russian	2 (5%)	2 (6%)	0 (0%)		
	Other	6 (15%)	6 (18%)	0 (0%)		
Marital status	Not married	6 (15%)	6 (18%)	0 (0%)	0.26	Pearson’s chi-squared
	Married	34 (85%)	28 (82%)	6 (100%)		
Education	Higher education	6 (15%)	6 (18%)	0 (0%)	0.12	Pearson’s chi-squared
	Secondary education	25 (62%)	19 (56%)	6 (100%)		
	Secondary vocational education	9 (22%)	9 (26%)	0 (0%)		
Family economic status	Low-income families	5 (12%)	5 (15%)	0 (0%)	0.36	Pearson’s chi-squared
	Middle-income families	31 (78%)	25 (74%)	6 (100%)		
	High-income families	4 (10%)	4 (12%)	0 (0%)		
Menstrual history	Regular	40 (100%)	34 (100%)	6 (100%)		Pearson’s chi-squared
Concomitant illness	No illness	4 (10%)	3 (9%)	1 (17%)	0.62	Pearson’s chi-squared
	Cardiovascular	2 (5%)	2 (6%)	0 (0%)		
	Nervous system	1 (2%)	1 (3%)	0 (0%)		
	Genitourinary	3 (8%)	3 (9%)	0 (0%)		
	Digestive	1 (2%)	1 (3%)	0 (0%)		
	Endocrine	4 (10%)	4 (12%)	0 (0%)		
	Veins and thrombosis	3 (8%)	3 (9%)	0 (0%)		
	Blood diseases	3 (8%)	3 (9%)	0 (0%)		
	Other	3 (8%)	3 (9%)	0 (0%)		
	Combined	16 (40%)	11 (32%)	5 (83%)		
Gynecological illness	No illness	20 (50%)	19 (56%)	1 (17%)	0.3	Pearson’s chi-squared
	Inflammatory diseases	10 (25%)	8 (24%)	2 (33%)		
	Non-inflammatory diseases	6 (15%)	4 (12%)	2 (33%)		
	Benign tumors	4 (10%)	3 (9%)	1 (17%)		
Gynecological surgeries	No surgery	26 (65%)	22 (65%)	4 (67%)	0.89	Pearson’s chi-squared
	Surgery on uterine body	11 (28%)	9 (26%)	2 (33%)		
	Surgery on cervix	2 (5%)	2 (6%)	0 (0%)		
	Surgery on ovaries	1 (2%)	1 (3%)	0 (0%)		
Pregnancy registration	Not registered for pregnancy follow-up	1 (2%)	1 (3%)	0 (0%)	0.37	Pearson’s chi-squared
	Before 12 weeks of gestation	33 (82%)	29 (85%)	4 (67%)		
	After 12 weeks of gestation	6 (15%)	4 (12%)	2 (33%)		
Preconception preparation	No preconception counseling	18 (45%)	14 (41%)	4 (67%)	0.25	Pearson’s chi-squared
	Yes	22 (55%)	20 (59%)	2 (33%)		
Visits	4 and more	25 (62%)	22 (65%)	3 (50%)	0.29	Pearson’s chi-squared
	3	2 (5%)	2 (6%)	0 (0%)		
	2	3 (8%)	2 (6%)	1 (17%)		
	1	4 (10%)	2 (6%)	2 (33%)		
	No data	5 (12%)	5 (15%)	0 (0%)		
	No visits	1 (2%)	1 (3%)	0 (0%)		
Folic acid supplementation	Did not take	16 (40%)	13 (38%)	3 (50%)	0.53	Pearson’s chi-squared
	Yes	18 (45%)	15 (44%)	3 (50%)		
	Missing data	6 (15%)	6 (18%)	0 (0%)		
Smoking	No	38 (95%)	32 (94%)	6 (100%)	0.54	Pearson’s chi-squared
	Yes	2 (5%)	2 (6%)	0 (0%)		
Alcohol use	No	40 (100%)	34 (100%)	6 (100%)		Pearson’s chi-squared
Hypertension	No	36 (90%)	30 (88%)	6 (100%)	0.38	Pearson’s chi-squared
	Yes	4 (10%)	4 (12%)	0 (0%)		
Antihypertensive medication use	No	36 (90%)	30 (88%)	6 (100%)	0.38	Pearson’s chi-squared
	Yes	4 (10%)	4 (12%)	0 (0%)		
Pre-eclampsia	No	32 (80%)	26 (76%)	6 (100%)	0.18	Pearson’s chi-squared
	Yes	8 (20%)	8 (24%)	0 (0%)		
Diabetes	No	37 (92%)	31 (91%)	6 (100%)	0.45	Pearson’s chi-squared
	Yes	3 (8%)	3 (8%)	0 (0%)		
Diabetes treatment	No treatment	37 (92%)	31 (91%)	6 (100%)	0.75	Pearson’s chi-squared
	Diet	2 (5%)	2 (6%)	0 (0%)		
	Insulin therapy	1 (2%)	1 (3%)	0 (0%)		
Thyroid disease	No	35 (88%)	30 (88%)	5 (83%)	0.74	Pearson’s chi-squared
	Yes	5 (12%)	4 (12%)	1 (17%)		
Cholestatic syndrome	No	39 (98%)	33 (97%)	6 (100%)	0.67	Pearson’s chi-squared
	Yes	1 (2%)	1 (3%)	0 (0%)		
Fetal diseases	No	27 (68%)	23 (68%)	4 (67%)	0.28	Pearson’s chi-squared
	Fetal IUGR	5 (12%)	3 (9%)	2 (33%)		
	Fetal defects	6 (15%)	6 (18%)	0 (0%)		
	Placental disorder	2 (5%)	2 (6%)	0 (0%)		
Oligohydramnios	No	33 (82%)	30 (88%)	3 (50%)	0.023	Pearson’s chi-squared
	Yes	7 (18%)	4 (12%)	3 (50%)		
Polyhydramnios	No	38 (95%)	32 (94%)	6 (100%)	0.54	Pearson’s chi-squared
	Yes	2 (5%)	2 (6%)	0 (0%)		
Chorioamnionitis	No	40 (100%)	34 (100%)	6 (100%)		Pearson’s chi-squared
Placental pathology	No	36 (90%)	30 (88%)	6 (100%)	0.38	Pearson’s chi-squared
	Premature separation of a normally located placenta	4 (10%)	4 (12%)	0 (0%)		
	Knew	37 (92%)	31 (91%)	6 (100%)		
Delivery	Preterm	25 (62%)	20 (59%)	5 (83%)	0.25	Pearson’s chi-squared
	Term	15 (38%)	14 (41%)	1 (17%)		
Vaginal delivery	Vaginal labor resulted with cesarean section	9 (22%)	9 (26%)	0 (0%)	0.11	Pearson’s chi-squared
	Induced labor	18 (45%)	16 (47%)	2 (33%)		
	Spontaneous labor	13 (32%)	9 (26%)	4 (67%)		
Cesarean section		31 (78%)	25 (74%)	6 (100%)	0.15	Pearson’s chi-squared
Levels of medical care	Level III	40 (100%)	34 (100%)	6 (100%)		Pearson’s chi-squared
Level of suboptimal help	II Level	21 (52%)	16 (47%)	5 (83%)	0.1	Pearson’s chi-squared
	III Level	19 (48%)	18 (53%)	1 (17%)		

**Table 3:** Perinatal mortality categories.

Category	Definition	Mortality Cases	
		Antenatal and Intranatal (%)	Neonatal (%)
**0**	Inadequacy of medical care was not identified	0	0
**1**	Inadequate medical care; a different management strategy would not have changed the outcome	0	11
**2**	Inadequate medical care; a different management strategy could have affected the outcome	52	59
**3**	Inadequate medical care; it would be reasonable to expect that a different management strategy might have affected the outcome	48	30

**Table 4:** Stratification of neonatal mortality cases.

Factor	Value	Intraventricular Hemorrhage		*p*-Value	Test	Sepsis		*p*-Value	Test
		No	Yes			No	Yes		
Gender				0.21	Pearson’s chi-squared			0.88	Pearson’s chi-squared
male	48 (63%)	31 (69%)	17 (55%)			37 (63%)	11 (65%)		
female	28 (37%)	14 (31%)	14 (45%)			22 (37%)	6 (35%)		
Birth weight, median (IQR)	1016.0 (748.0, 1575.0)	1186.0 (813.0, 1950.0)	890.0 (603.0, 1270.0)	0.022	Wilcoxon rank-sum	1041.0 (756.0, 1460.0)	857.0 (740.0, 1600.0)	0.63	Wilcoxon rank-sum
Gestational age (week), median (IQR)	27.0 (25.5, 30.0)	29.0 (26.0, 32.0)	26.0 (25.0, 28.0)	0.003	Wilcoxon rank-sum	27.0 (26.0, 30.0)	27.0 (25.0, 30.0)	0.67	Wilcoxon rank-sum
Small for gestational age				0.32	Pearson’s chi-squared			0.72	Pearson’s chi-squared
no	65 (86%)	37 (82%)	28 (90%)			50 (85%)	15 (88%)		
yes	11 (14%)	8 (18%)	3 (10%)			9 (15%)	2 (12%)		
Maturity				0.15	Pearson’s chi-squared			0.85	Pearson’s chi-squared
preterm	66 (87%)	37 (82%)	29 (94%)			51 (86%)	15 (88%)		
full-term	10 (13%)	8 (18%)	2 (6%)			8 (14%)	2 (12%)		
Antenatal corticosteroid therapy				0.72	Pearson’s chi-squared			0.95	Pearson’s chi-squared
not carried out/incomplete course	37 (49%)	24 (53%)	13 (42%)			28 (47%)	9 (53%)		
carried out	20 (26%)	12 (27%)	8 (26%)			15 (25%)	5 (29%)		
missing data	19 (25%)	9 (20%)	10 (32%)			16 (27%)	3 (18%)		
Maternal antibiotic prophylaxis				0.67	Pearson’s chi-squared			0.17	Pearson’s chi-squared
no	40 (53%)	23 (51%)	17 (55%)			34 (58%)	6 (35%)		
yes	32 (42%)	20 (44%)	12 (39%)			23 (39%)	9 (53%)		
missing	4 (5%)	2 (4%)	2 (6%)			2 (3%)	2 (12%)		
Level of suboptimal help				0.94	Pearson’s chi-squared			0.84	Pearson’s chi-squared
I Level	8 (11%)	5 (11%)	3 (10%)			6 (10%)	2 (12%)		
II Level	45 (59%)	27 (60%)	18 (58%)			36 (61%)	9 (53%)		
III Level	23 (30%)	13 (29%)	10 (32%)			17 (29%)	6 (35%)		
Patent ductus arteriosus				0.006	Pearson’s chi-squared			0.091	Pearson’s chi-squared
no + no Echo	30 (39%)	16 (36%)	14 (45%)			26 (44%)	4 (24%)		
yes	24 (32%)	10 (22%)	14 (45%)			15 (25%)	9 (53%)		
no	22 (29%)	19 (42%)	3 (10%)			18 (31%)	4 (24%)		
Echocardiogram				0.40	Pearson’s chi-squared			0.13	Pearson’s chi-squared
no	30 (39%)	16 (36%)	14 (45%)			26 (44%)	4 (24%)		
yes	46 (61%)	29 (64%)	17 (55%)			33 (56%)	13 (76%)		
Confirmed sepsis				0.60	Pearson’s chi-squared				
no	59 (78%)	34 (76%)	25 (81%)						
yes	17 (22%)	11 (24%)	6 (19%)						
Death day, median (IQR)	2.0 (1.0, 4.0)	2.0 (1.0, 4.0)	2.0 (1.0, 4.0)	0.50	Wilcoxon rank-sum	2.0 (1.0, 4.0)	3.0 (1.0, 5.0)	0.14	Wilcoxon rank-sum
**Total**	**76**	**45**	**31**			**59**	**17**		

## Data Availability

Raw data related to this study is available per reasonable request.

## Supplementary Materials

The following supporting information can be downloaded at: https://www.mdpi.com/article/10.3390/medsci13020077/s1, Figure S1: “Factors associated with antenatal and perinatal mortality”; Figure S2: “Factors associated with neonatal mortality”.
