# Supplementary material for: Confidential Audit of Perinatal Mortality in the Republic of Kazakhstan: A Pilot Study
Source: Med Sci (Basel). 2025 Jun 13;13(2):77. doi: 10.3390/medsci13020077 (PMC12194885; doi:10.3390/medsci13020077)
Supplement: Supplementary file 1 [file medsci-13-00077-s001.zip › medsci-3695539-supplementary.pdf]

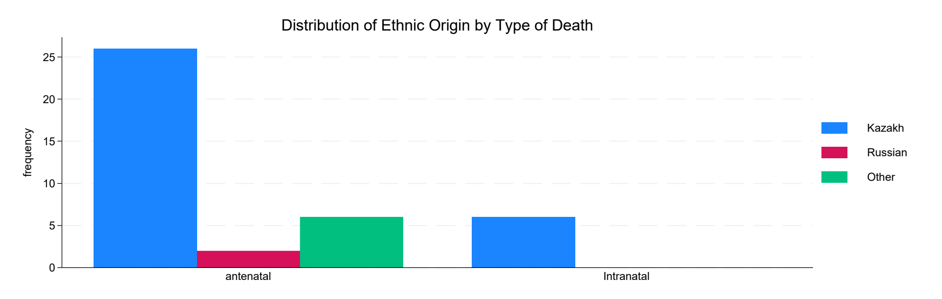
Distribution of Ethnic Origin by Type of Death

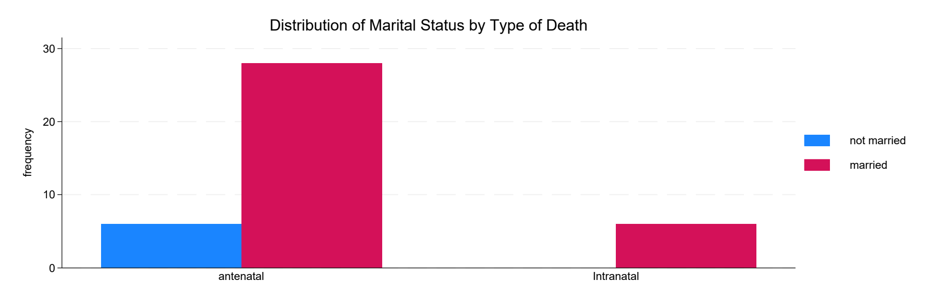
Distribution of Marital Status by Type of Death

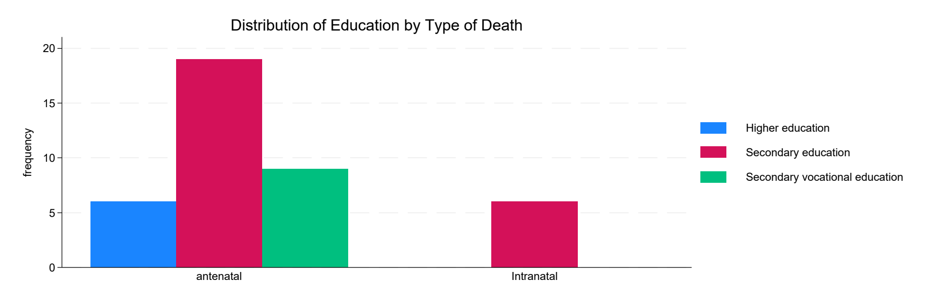
Distribution of Education by Type of Death

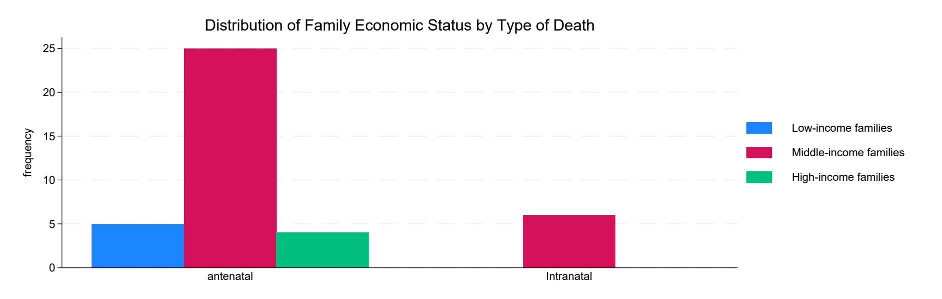
Distribution of Family Economic Status by Type of Death

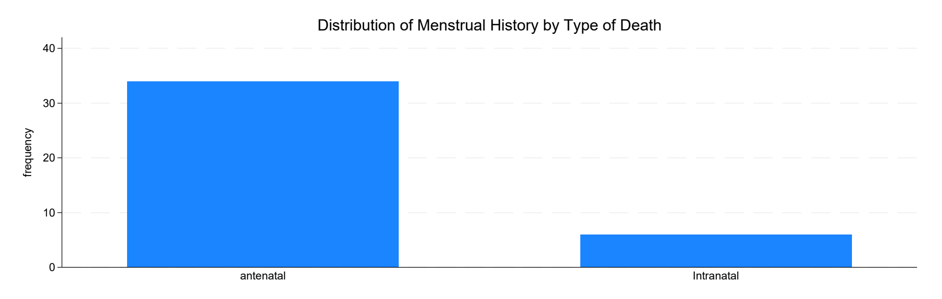
Distribution of Menstrual History by Type of Death

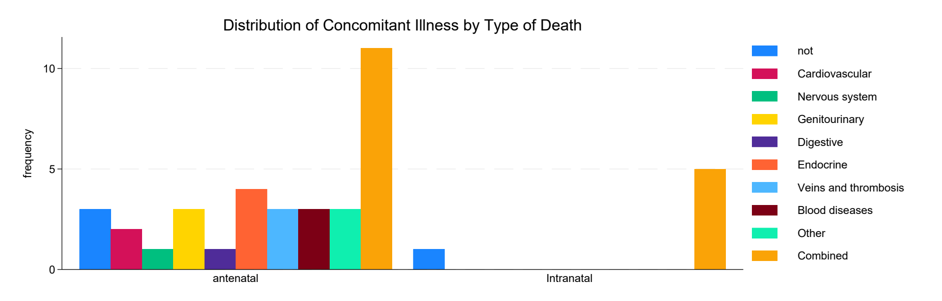
Distribution of Concomitant Illness by Type of Death

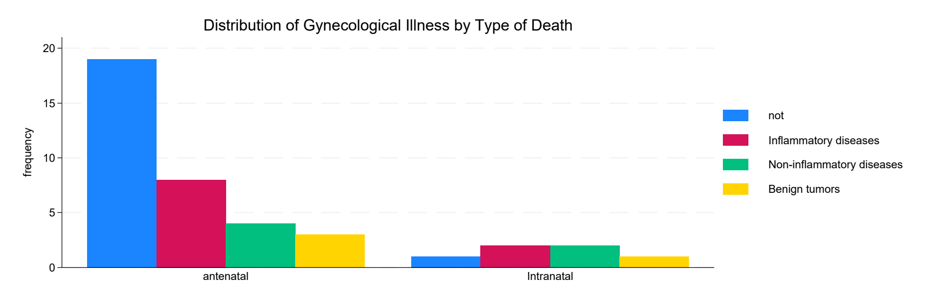
Distribution of Gynecological Illness by Type of Death

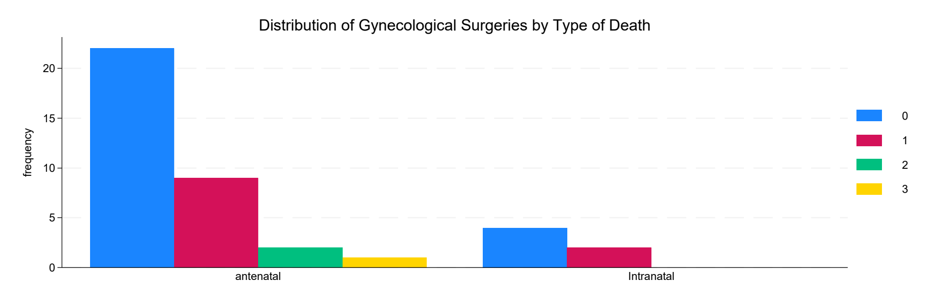
Distribution of Gynecological Surgeries by Type of Death

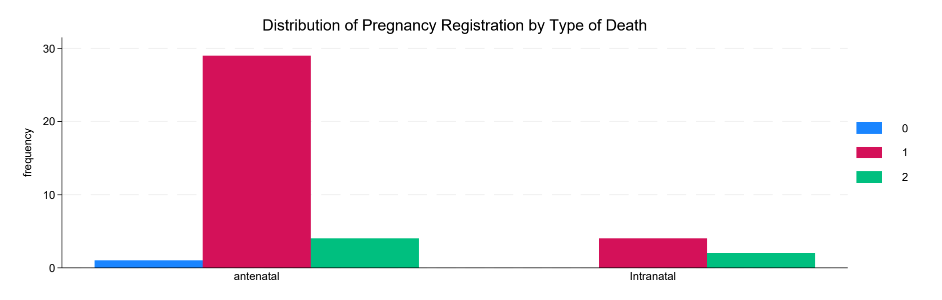
Distribution of Pregnancy Registration by Type of Death

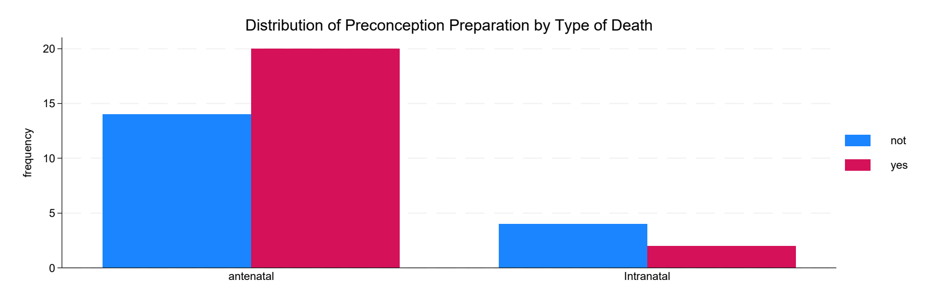
Distribution of Preconception Preparation by Type of Death

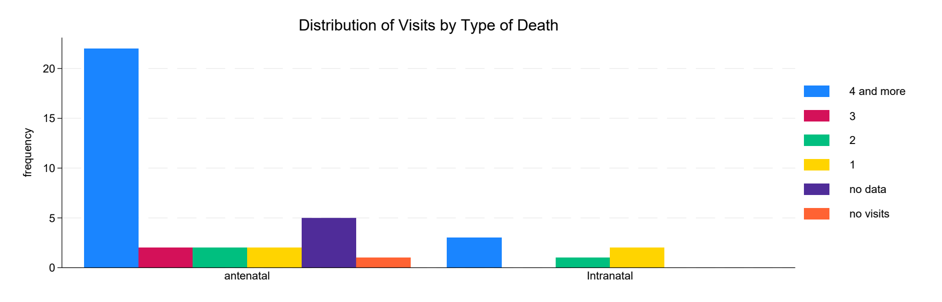
Distribution of Visits by Type of Death

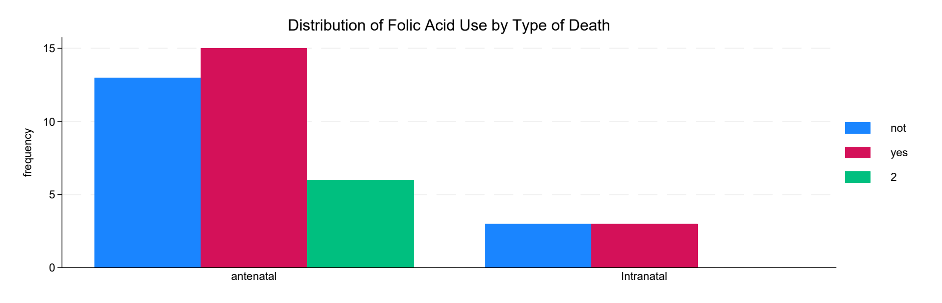
Distribution of Folic Acid Use by Type of Death

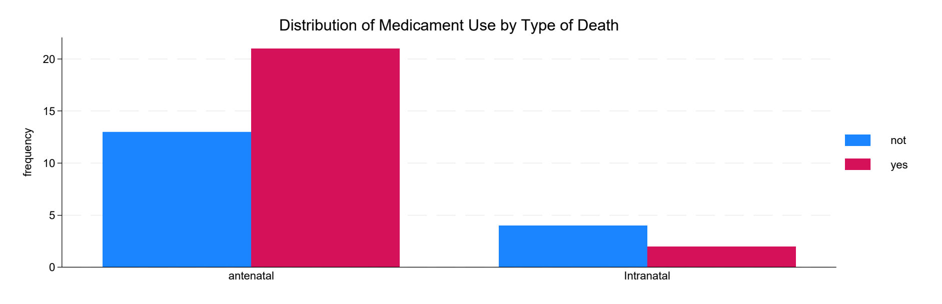
Distribution of Medicament Use by Type of Death

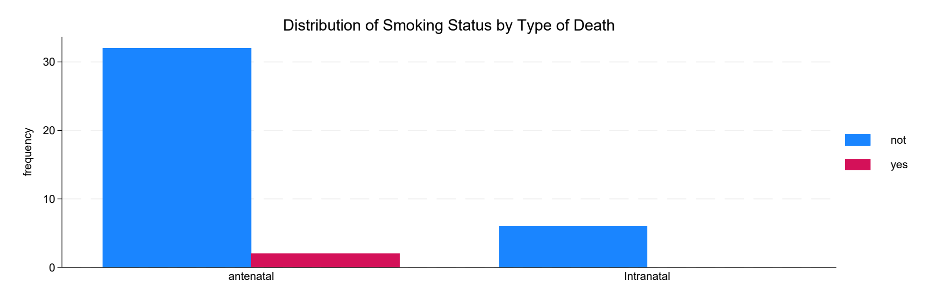
Distribution of Smoking Status by Type of Death

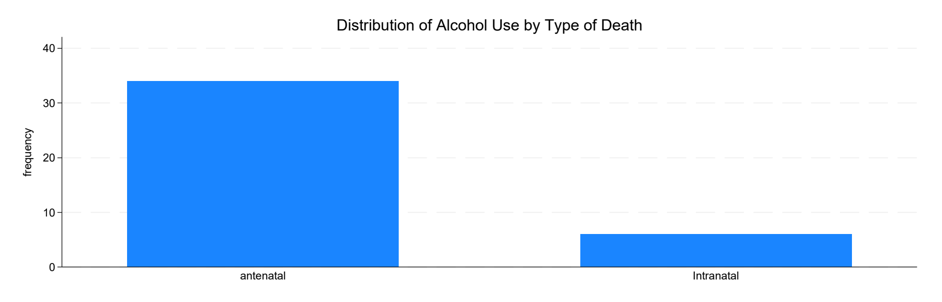
Distribution of Alcohol Use by Type of Death

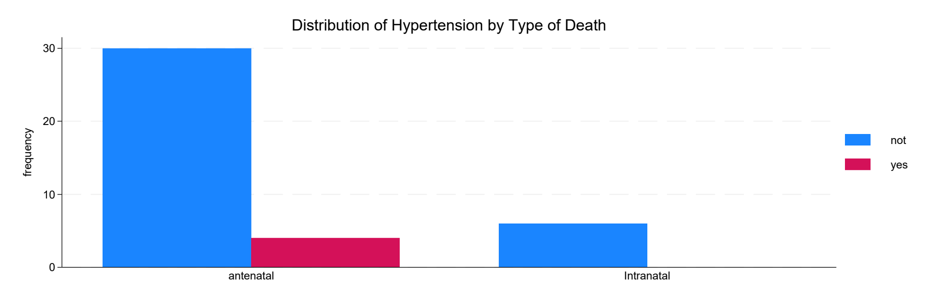
Distribution of Hypertension by Type of Death

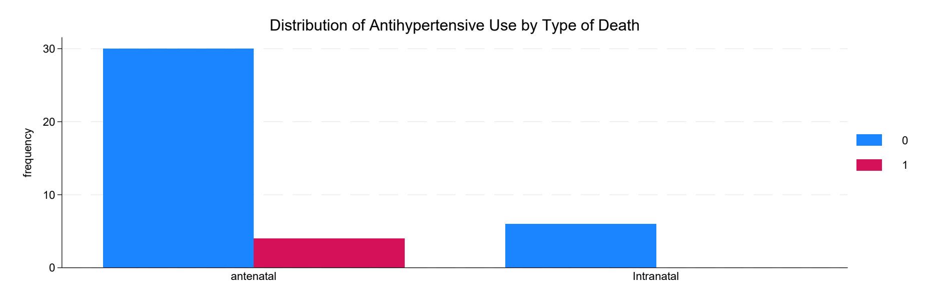
Distribution of Antihypertensive Use by Type of Death

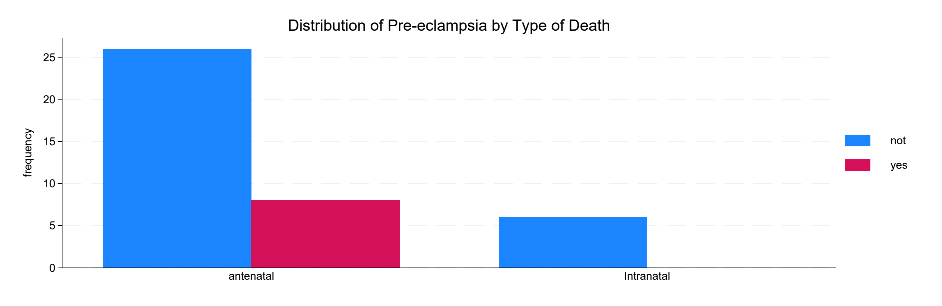
Distribution of Pre-eclampsia by Type of Death

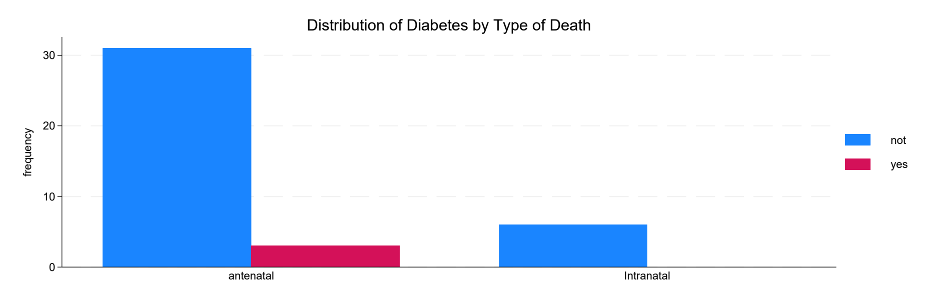
Distribution of Diabetes by Type of Death

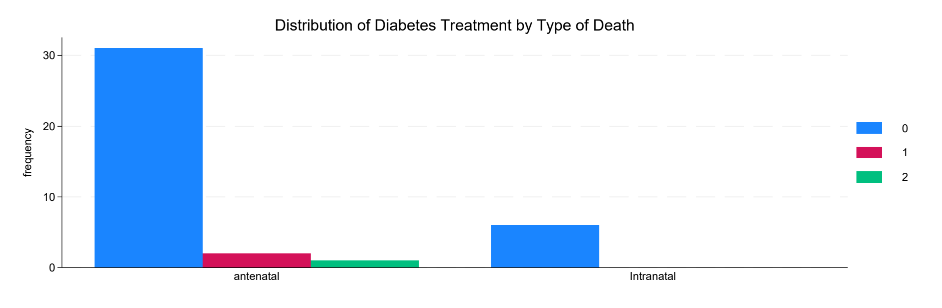
Distribution of Diabetes Treatment by Type of Death

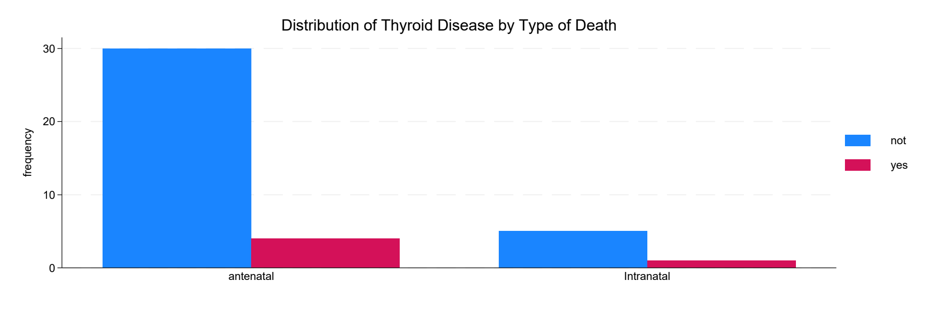
Distribution of Thyroid Disease by Type of Death

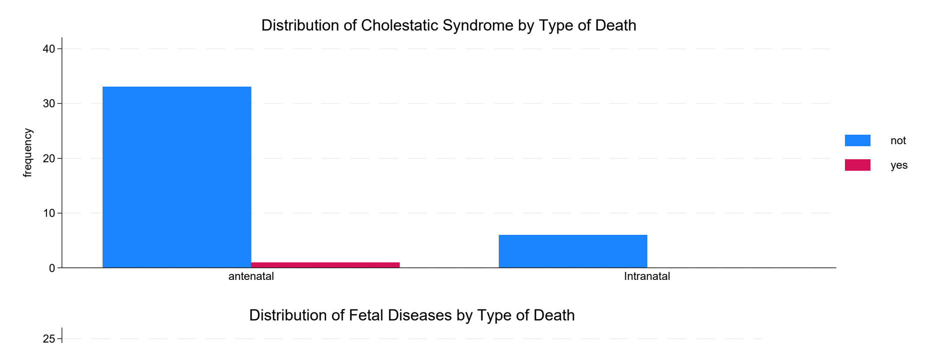
Distribution of Cholestatic Syndrome by Type of Death

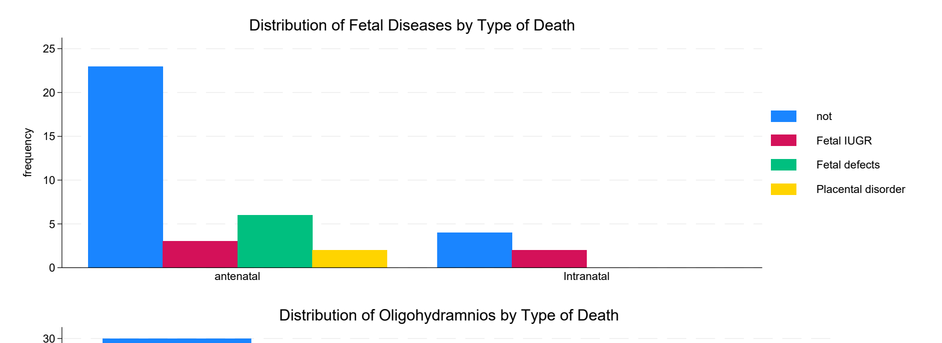
Distribution of Fetal Diseases by Type of Death

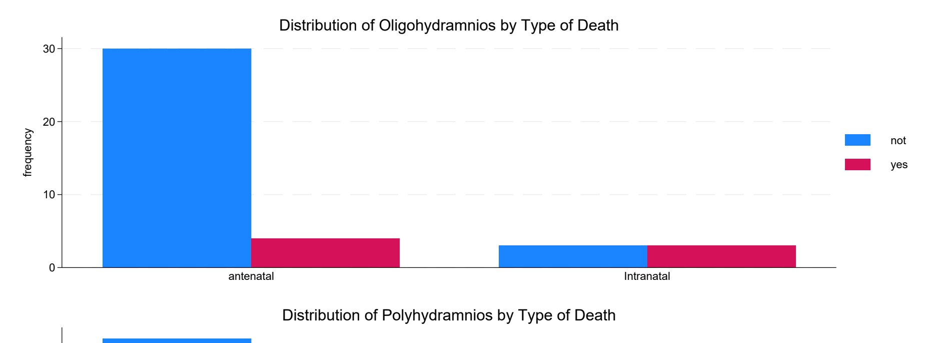
Distribution of Oligohydramnios by Type of Death

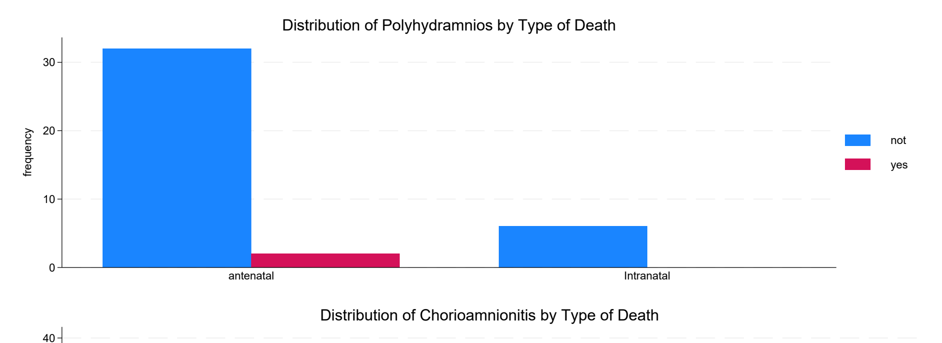
Distribution of Polyhydramnios by Type of Death

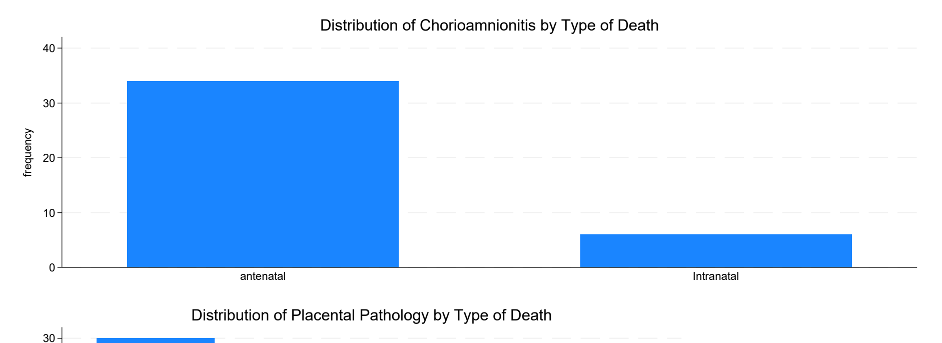
Distribution of Chorioamnionitis by Type of Death

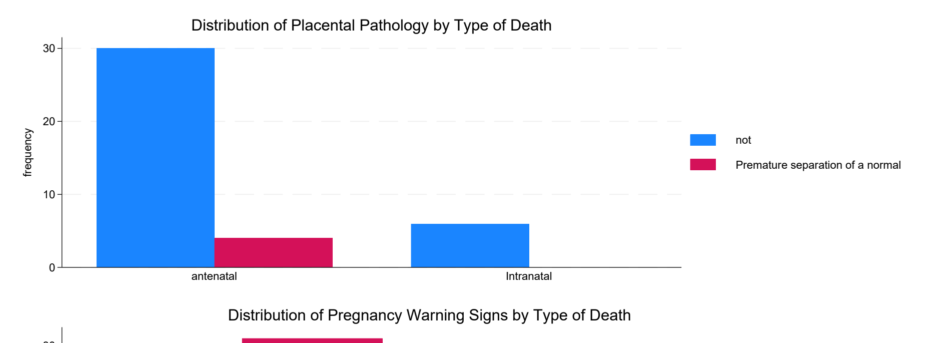
Distribution of Placental Pathology by Type of Death

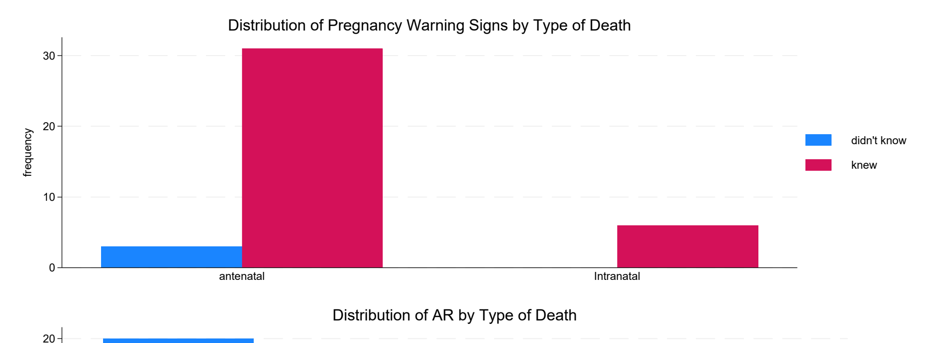
Distribution of Pregnancy Warning Signs by Type of Death

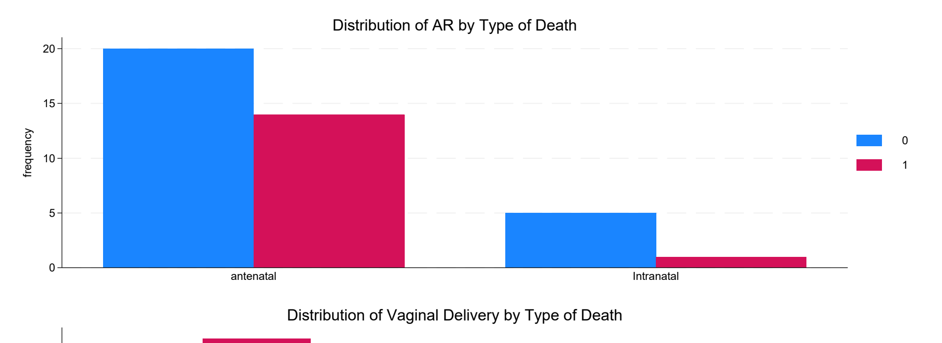
Distribution of AR by Type of Death

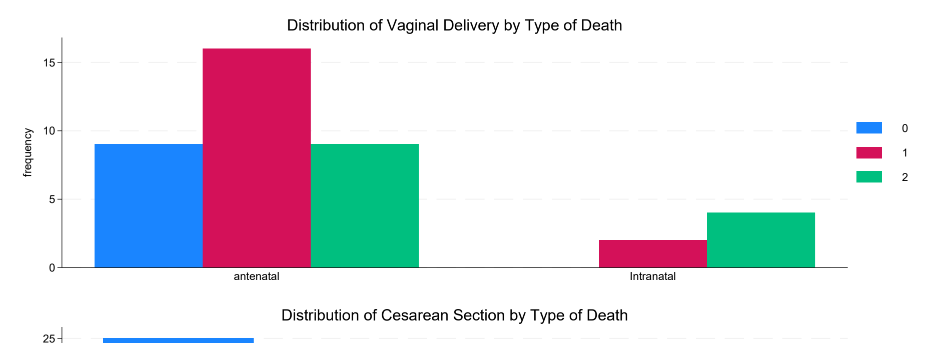
Distribution of Vaginal Delivery by Type of Death

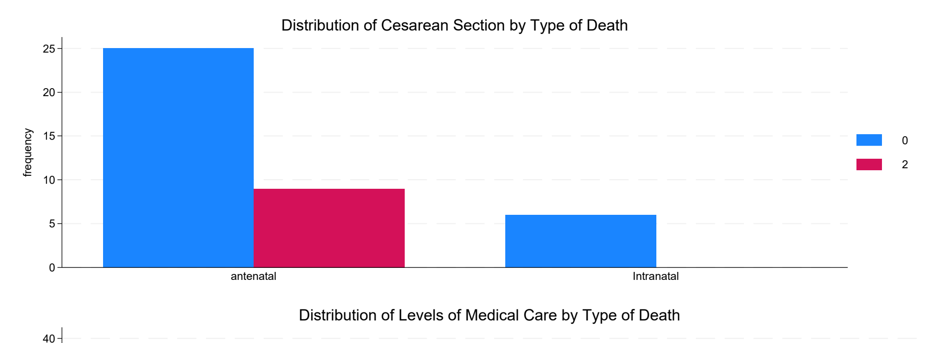
Distribution of Cesarean Section by Type of Death

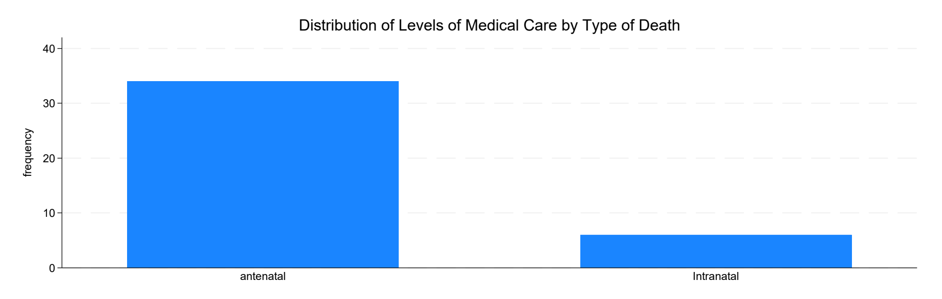
Distribution of Levels of Medical Care by Type of Death

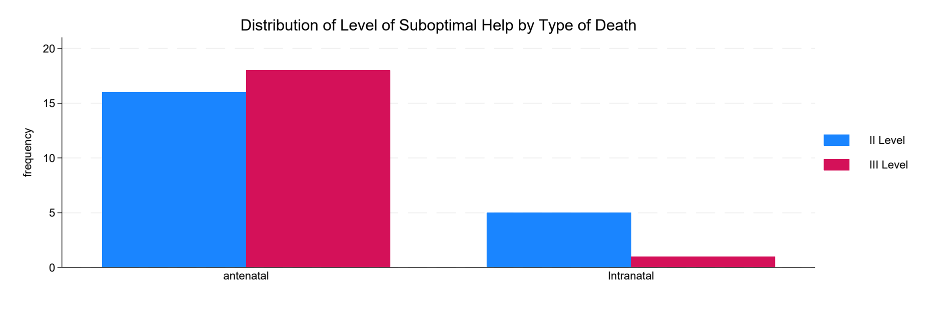
Distribution of Level of Suboptimal Help by Type of Death
